# Association Between Cognitive Dysfunction, TYG Index, and Depression in Older Adults: Based on the NHANES Database, 2011–2014

**DOI:** 10.1002/brb3.70824

**Published:** 2025-09-30

**Authors:** Qinghua Guo, Chao Chen, Libo Guo, Yong Wang, Shaomei Shang

**Affiliations:** ^1^ Outpatient Department Peking University Sixth Hospital；Peking University Institute of Mental Health; NHC Key Laboratory of Mental Health (Peking University); National Clinical Research Centerfor Mental Disorders (Peking University Sixth Hospital) Beijing China; ^2^ School of Nursing Peking University Beijing China; ^3^ Department of Nursing Peking University Sixth Hospital；Peking University Institute of Mental Health; NHC Key Laboratory of Mental Health (Peking University); National Clinical Research Centerfor Mental Disorders (Peking University Sixth Hospital) Beijing China

## Abstract

**Background:**

The relationship between cognitive impairment, triglyceride‐glucose (TyG) index, and depression in the elderly remains unclear. This study aims to explore the associations among cognitive impairment, TyG index, and the risk of depression in older adults, providing a basis for targeted prevention strategies.

**Methods:**

This cross‐sectional study utilized data from the National Health and Nutrition Examination Survey (NHANES) from 2011 to 2014. Depression was assessed using the Patient Health Questionnaire‐9 (PHQ‐9). Cognitive impairment was defined as the lowest quartile of three cognitive tests: the Consortium to Establish a Registry for Alzheimer's Disease (CERAD) test for learning and memory, the Animal Fluency test for executive function, and the Digit Symbol Substitution Test (DSST) for attention and processing speed. The TyG index was calculated as ln[triglycerides (mg/dL) × fasting glucose (mg/dL)/2], and participants were categorized into quartiles based on their TyG index. Multivariable logistic regression models were employed to investigate the relationships between cognitive impairment, TyG index, and depression in the elderly.

**Results:**

A total of 2042 elderly participants (aged ≥ 60 years) were included in the study, among whom 312 (15.3%) were diagnosed with depression. Both cognitive impairment and higher TyG index were significantly associated with increased depressive symptoms among older adults in the United States. The risk of depression was 2.64 times higher (95% confidence interval [CI]: 1.33–5.23) in those with cognitive impairment compared to those with normal cognitive function. Participants in the highest TyG quartile had a multivariable‐adjusted odds ratio (OR) of 1.61 (95% CI: 1.10–2.35) for depression compared to those in the lowest quartile. Similar results were observed across different genders, age groups, and baseline comorbidities.

**Conclusion:**

Our findings suggest that higher TyG index and cognitive impairment (including deficits in learning and memory, executive function, and attention/processing speed) are associated with a greater likelihood of depressive symptoms in older adults.

## Graphical abstract

Higher TyG index and cognitive impairment are independently associated with an increased risk of depression in older adults. These findings highlight the value of integrating metabolic and cognitive screening to improve early identification and prevention strategies for late‐life depression.

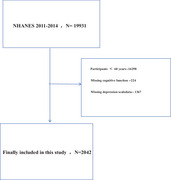

**Abbreviations**
AA: associate of arts
APOE‐e4: apolipoprotein E epsilon 4 allele
BDNF: brain‐derived neurotrophic factor
BMI: body mass index
CDC: Centers for Disease Control and Prevention
CERAD: Consortium to Establish a Registry for Alzheimer's Disease
CI: confidence interval
DSM‐V: Diagnostic and Statistical Manual of Mental Disorders, Fifth Edition
DSST: Digit Symbol Substitution Test
GED: general educational development
IL‐6: interleukin‐6
MAR: missing at random
MCI: mild cognitive impairment
MICE: multiple imputation by chained equations
NHANES: National Health and Nutrition Examination Survey
OR: odds ratio
PHQ‐9: Patient Health Questionnaire‐9
SD: standard deviation
TGF‐β1: transforming growth factor Beta 1
TNF‐α: tumor necrosis factor alpha
TyG: triglyceride‐glucose

## 1 Introduction

Depression is a common mood disorder characterized by persistent sadness, hopelessness, and feelings of worthlessness, along with a lack of interest in previously enjoyable activities. These symptoms significantly impair social and psychological functioning and reduce the quality of life (Thapar et al. 2022). Depression is a major contributor to the burden of mental health‐related diseases and global disability (GBD 2019 Diseases and Injuries Collaborators 2020). According to the World Health Organization (WHO), approximately 280 million people worldwide were affected by depression in 2019, and this number is projected to rise to 350 million by 2025 (WHO 2023). Among individuals aged 60 and above, the prevalence of depression ranges from 10% to 20%, and this trend is increasing (Monroe and Harkness 2022; Sjöberg et al. 2017; Zhao et al. 2024). Meta‐analyses have identified several risk factors for depression in the elderly, including female gender, age over 60, insomnia, low educational background, smoking, physical illnesses such as diabetes, hypertension, heart disease, stroke, head injuries, poor sleep quality, and cognitive impairment (Nguyen et al. 2024).

Cognitive functions, including learning, attention, memory, and decision‐making, are crucial for maintaining the quality of life in healthy older adults (Dumas 2017). Research indicates that depression, particularly in the elderly, is often associated with cognitive impairments, especially memory decline (Morimoto et al. 2015). Therefore, we focus on exploring the relationship between cognitive impairment and depression in older adults. Previous studies have shown that individuals with mild cognitive impairment (MCI) are more likely to develop depression (Ma 2020). Furthermore, those with both MCI and depression typically exhibit slower processing speeds and impairments in executive function, flexibility, and verbal fluency (Ma 2020). These findings suggest a potential link between cognitive impairment and depression in the elderly. The bidirectional relationship between cognitive impairment and depression may share common biological mechanisms, including vascular diseases, alterations in glucocorticoid steroid levels, hippocampal atrophy, increased amyloid‐beta plaque deposition, inflammatory changes, and deficits in neurotrophic factors (Byers and Yaffe 2011).

There is an important two‐way link between diabetes and depression over the long term, with one condition doubling the risk of the other (Krupa et al. 2024). Additionally, insulin resistance, a key feature of type 2 diabetes, hypertension, and cardiovascular diseases, has been linked to depression in observational studies (Fernandes et al. 2022; Chatterjee et al. 2016, Abeysekera et al. 2024), particularly through the measurement of the triglyceride‐glucose (TyG) index, a novel marker for assessing insulin resistance. Despite this premise, the association between TYG and depression has not been fully researched and therefore remains controversial (Behnoush et al. 2024; Shi et al. 2021). Other factors such as gender differences, for example, may influence this association (Behnoush et al. 2024). A meta‐analysis of studies has shown that a high TyG index is associated with depression and that a 1‐unit increase in TyG index is significantly associated with a 75.0% increase in the odds of MDD (odds ratio [OR] 1.750, 95% confidence interval [CI] 1.284–2.384, *p* < 0.001) (Behnoush et al. 2024). Although the TyG index has been associated with various health issues, including cardiovascular diseases and dementia (Liu et al. 2020), its relationship with depression in the elderly remains unclear.

Recent meta‐analyses have consistently demonstrated the clinical utility of the TyG index as a comprehensive marker for assessing cardiometabolic risks (Nayak et al. 2024). Specifically, elevated TyG levels have been significantly associated with increased risks of heart failure (HR = 1.21, 95% CI: 1.12–1.30), atrial fibrillation (SMD = 1.22, 95% CI: 0.57–1.87), type 2 diabetes mellitus (RR = 3.53, 95% CI: 2.74–4.54), obstructive sleep apnea (SMD = 0.86, 95% CI: 0.57–1.15), peripheral arterial disease, chronic kidney disease (RR = 1.46, 95% CI: 1.32–1.63), dementia (OR = 1.14, 95% CI: 1.12–1.16), and ischemic stroke (OR = 1.37, 95% CI: 1.22–1.54) (Nayak et al. 2024). These findings highlight the potential of the TyG index as a systemic metabolic marker not only for insulin resistance but also for multiple cardiometabolic disorders, reinforcing its value in comprehensive clinical risk stratification and preventive interventions.

Based on this, we hypothesize that cognitive impairment and TyG index are associated with depression in older adults. Higher TyG index and cognitive impairment (across overall cognition, learning and memory, executive function, attention, and processing speed) may indicate a higher risk of severe depressive symptoms. This study will analyze data from the large, nationally representative National Health and Nutrition Examination Survey (NHANES) database to quantify the relationship between cognitive impairment, TyG index, and depression in the elderly, aiming to develop prevention strategies to improve the quality of life in this population.

## 2 Methods

### 2.1 Study Population

The NHANES (https://wwwn.cdc.gov/nchs/nhanes/Default.aspx) aims to assess the health and nutritional status of adults and children in the United States. It uses a multistage sampling method, combining questionnaires, physical examinations, and laboratory tests to obtain a nationally representative sample. The NHANES study is reviewed by the Research Ethics Review Board of the National Center for Health Statistics, with approvals obtained semiannually between full proposal reviews. Informed consent was obtained from all participants.

**FIGURE 1:**
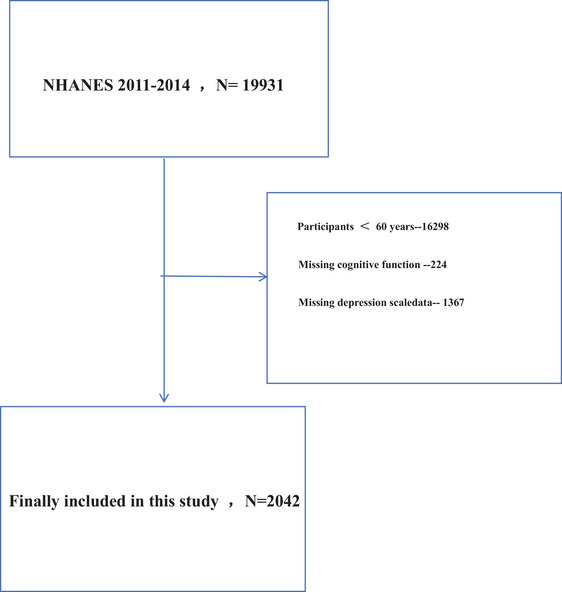
Flow chart for participants recruitment, NHANES 2011–2014. The participant selection process from the initial NHANES dataset (*n* = 19,931), including exclusion criteria such as age < 60, missing cognitive data, or missing depression scores, ultimately resulting in 2042 eligible older adults.

This study utilized a cross‐sectional design, collecting information through standardized interviews, physical examinations, and biospecimen tests. Participants from two NHANES cycles (2011–2014) were included, excluding individuals under 60 years of age. A total of 2042 participants completed the Patient Health Questionnaire‐9 (PHQ‐9) for depression symptoms and cognitive function tests, including word learning and recall modules from the Consortium to Establish a Registry for Alzheimer's Disease (CERAD), the Animal Fluency test, and the Digit Symbol Substitution Test (DSST). Detailed information is provided in Figure 1. Missing information was imputed using polynomial regression and logistic regression. Missing covariate data (approximately 6.7%) were addressed by multiple imputation by chained equations (MICE), which assumes a missing‐at‐random (MAR) mechanism. Twenty imputed datasets were generated using predictive mean matching for continuous variables and logistic regression for categorical variables. Sensitivity analyses comparing the results from complete‐case analysis (participants with complete data only) and the multiply imputed datasets revealed no significant differences or biases, indicating robustness of the findings to different missing‐data approaches.

### 2.2 Data Measurement

#### 2.2.1 Outcome Ascertainment: Depressive Symptoms

Depressive symptoms were assessed using the PHQ‐9, a valid measure based on the Diagnostic and Statistical Manual of Mental Disorders (DSM)‐V criteria. The PHQ‐9 scores nine items from “0” (not at all) to “3” (nearly every day). The total score, ranging from 0 to 27, represents the severity of depressive symptoms. A PHQ‐9 score ≥ 10 is defined as depressive symptoms, with a sensitivity of 88% and specificity of 88% (Costantini et al. 2021; Levis et al. 2019). Participants were categorized into no depressive symptoms (PHQ‐9 score < 10) and depressive symptoms (PHQ‐9 score ≥ 10).

#### 2.2.2 Exposure Measurement: Cognitive Function

Only individuals aged 60 and above participated in the survey. Cognitive function was assessed across four dimensions. The CERAD Word Learning Test evaluated the ability to learn new verbal information, including immediate and delayed recall. The Animal Fluency Test assessed categorical verbal fluency in executive function. The DSST evaluated processing speed, sustained attention, and working memory. Cognitive impairment was defined based on performance across the four cognitive dimensions. Participants scoring in the lowest quartile (bottom 25%) were assigned a score of 1, indicating impairment, while those in higher quartiles were assigned a score of 0, indicating normal cognitive function (Chen et al. 2017).

##### 2.2.2.1 Assessment of TyG Index

The TyG index was calculated as TyG index = Ln [fasting TG (mg/dL) × fasting glucose (mg/dL)/2]. Triglycerides and fasting glucose were measured using enzymatic methods on the Roche Modular P and Roche Cobas 6000 analyzers. Fasting glucose was measured using a hexokinase‐mediated reaction on the Roche/Hitachi Cobas C 501 analyzer.

#### 2.2.3 Covariate Assessment

Covariates included gender, age, race, education level, body mass index (BMI), alcohol consumption, smoking, hypertension, diabetes, sleep difficulties, and cardiovascular disease. Race categories were Mexican American, other Hispanic, non‐Hispanic White, non‐Hispanic Black, or other (including multiracial). Education levels were categorized as less than nineth grade, 9–11th grade, high school graduate/general educational development (GED), some college or associate of arts (AA) degree, and college graduate or above. BMI categories were < 25, 25–30, and > 30 kg m^−^^2^. Alcohol consumption was defined as drinking more than 12 times per year. Smoking was defined as having smoked at least 100 cigarettes in a lifetime. Hypertension, diabetes, and cardiovascular diseases (asthma, congestive heart failure, coronary heart disease, angina, heart attack, and stroke) were determined by self‐reported doctor diagnoses. Sleep difficulties were defined as self‐reported sleep problems diagnosed by a health professional.

### 2.3 Statistical Analysis

Data processing and analysis were performed using R version 4.3.0 (2023‐04‐21), along with Storm Statistical Platform (www.medsta.cn/software).Given the complex sampling design of NHANES, sample weights, clustering, and stratification were included in all analyses as required (Wu et al. 2023). Participants were categorized into two groups based on PHQ‐9 scores: no depressive symptoms (PHQ‐9 score < 10) and depressive symptoms (PHQ‐9 score ≥ 10).

Continuous variables were summarized as means and standard deviations (SD), while categorical variables were expressed as frequencies and percentages. One‐way ANOVA was used for continuous variables, and Pearson's chi‐square test was used for categorical variables to compare baseline characteristics between groups with and without depressive symptoms. Multivariable logistic regression models were developed to assess the relationship between cognitive function, TyG index, and depressive symptoms, including three models to control for confounders. Model 1 was unadjusted; Model 2 adjusted for age, race, gender, education, and BMI; and Model 3 further adjusted for smoking, drinking, hypertension, diabetes, sleep disorders, and cardiovascular diseases. Multiple imputation was used for missing covariate data. Stratified analyses were conducted by gender, race, education, BMI, hypertension, and diabetes. A *p*‐value < 0.05 was considered statistically significant.

### 2.4 Ethics Approval and Consent to Participate

The NHANES protocol was approved by the Institutional Review Board of the National Center for Health Statistics, Centers for Disease Control and Prevention (CDC). Written informed consent was obtained from each participant before participating in the study.

## 3 Results

### 3.1 Baseline Characteristics

The study included 2042 participants, comprising 312 individuals with depressive symptoms and 1730 without depressive symptoms (Table 1). Compared to those without depressive symptoms, participants with depressive symptoms had higher TyG index values; a higher proportion of females; a greater percentage of individuals with BMI > 30 kg m^−^^2^; and higher incidences of hypertension, diabetes, sleep disorders, and cardiovascular diseases (*p* < 0.05). Additionally, those with depressive symptoms exhibited lower scores in overall cognitive function, CERAD test, Animal Fluency test, and DSST (*p* < 0.05).

**TABLE 1:** Unweighted characteristics of the study population (age ≧ 60 years) based on with and without depressive symptoms, NHANES 2011–2014, USA.

Variables	Total (*n* = 2042)	Non‐depressive symptoms (PHQ < 10, %) (*n* = 1730)	Depressive symptoms (PHQ ≥ 10, %) (*n* = 312)	Statistic	*p*
Age, mean ± SD	69.69 ± 6.92	69.94 ± 6.92	68.31 ± 6.80	*t* = 3.82	**<0.001**
CERAD, mean ± SD	24.87 ± 7.19	25.15 ± 7.12	23.29 ± 7.40	*t* = 4.21	**<0.001**
Animal Fluency: score total, mean ± SD	16.23 ± 5.60	16.56 ± 5.60	14.42 ± 5.22	*t* = 6.17	**<0.001**
Digit Symbol: score, mean ± SD	45.29 ± 17.51	46.73 ± 17.26	36.88 ± 16.54	*t* = 8.92	**<0.001**
Cognitive function score total, mean ± SD	83.59 ± 28.12	85.90 ± 27.58	70.80 ± 27.67	*t* = 8.89	**<0.001**
TyG, mean ± SD	8.88 ± 0.70	8.86 ± 0.69	9.03 ± 0.76	*t* = ‐3.64	**<0.001**
Gender, *n* (%)				*χ* ^2^ = 3.71	0.054
Male	867 (42.46)	750 (43.35)	117 (37.50)		
Female	1175 (57.54)	980 (56.65)	195 (62.50)		
Race, *n* (%)				*χ* ^2^ = 43.64	**<0.001**
Mexican American	183 (8.96)	137 (7.92)	46 (14.74)		
Other Hispanic	224 (10.97)	170 (9.83)	54 (17.31)		
Non‐Hispanic White	978 (47.89)	864 (49.94)	114 (36.54)		
Non‐Hispanic Black	465 (22.77)	385 (22.25)	80 (25.64)		
Other race	192 (9.40)	174 (10.06)	18 (5.77)		
Education, *n* (%)				*χ* ^2^ = 86.02	**<0.001**
Less than nineth grade	254 (12.44)	177 (10.23)	77 (24.68)		
9–11th grade	317 (15.52)	245 (14.16)	72 (23.08)		
High school graduate/GED or equivalent	496 (24.29)	431 (24.91)	65 (20.83)		
Some college or AA degree	554 (27.13)	485 (28.03)	69 (22.12)		
College graduate or above	421 (20.62)	392 (22.66)	29 (9.29)		
BMI category, *n* (%)				*χ* ^2^ = 14.63	**<0.001**
< 25 kg/m^2^	522 (25.56)	457 (26.42)	65 (20.83)		
25–30 kg/m^2^	673 (32.96)	586 (33.87)	87 (27.88)		
> 30 kg/m^2^	847 (41.48)	687 (39.71)	160 (51.28)		
Smoking status, *n* (%)				*χ* ^2^ = 2.30	0.129
Yes	1044 (51.18)	872 (50.46)	172 (55.13)		
No	996 (48.82)	856 (49.54)	140 (44.87)		
Drinking status, *n* (%)				*χ* ^2^ = 0.04	0.849
Yes	331 (48.18)	277 (48.34)	54 (47.37)		
No	356 (51.82)	296 (51.66)	60 (52.63)		
Hypertension, *n* (%)				*χ* ^2^ = 4.06	**0.044**
Yes	1365 (66.98)	1142 (66.09)	223 (71.94)		
No	673 (33.02)	586 (33.91)	87 (28.06)		
Diabetes, *n* (%)				*χ* ^2^ = 27.80	**<0.001**
Yes	535 (26.23)	417 (24.12)	118 (37.94)		
No	1407 (68.97)	1222 (70.68)	185 (59.49)		
Sleeping disorders, *n* (%)				*χ* ^2^ = 35.74	**<0.001**
Yes	295 (14.48)	216 (12.51)	79 (25.48)		
No	1742 (85.52)	1511 (87.49)	231 (74.52)		
Cardiovascular diseases, *n* (%)				*χ* ^2^ = 14.93	**<0.001**
Yes	155 (26.50)	100 (22.52)	55 (39.01)		
No	430 (73.50)	344 (77.48)	86 (60.99)		

*Note*: Demographic and clinical characteristics comparison between participants with and without depressive symptoms, including gender, age, education, BMI, TyG index, cognitive test scores, and comorbidities. Continuous variables were presented as mean ± standard deviation, categorical variables were presented as numbers (percentages).

Abbreviations: BMI: body mass index, *χ* ^2^: chi‐square test, PHQ: Patient Health Questionnaire, SD: standard deviation, *t*: *t*‐test.

### 3.2 Cognitive Impairment Levels and TyG Associations With Risk of Depressive Symptoms

As shown in Table 2, cognitive impairment (overall cognitive function score, CERAD test, Animal Fluency test, and DSST) and TyG index were positively associated with the risk of depressive symptoms (Model 1). After adjusting for covariates (Models 2 and 3), the association between cognitive impairment and depressive symptoms became stronger. In the final model (Model 3), the OR for depressive symptoms was 2.64 (1.33, 5.23) compared to participants without depressive symptoms, with other final ORs being 2.16 (1.29, 3.63), 2.30 (1.41, 3.73), and 2.12 (1.26, 3.54). When TyG index was treated as a continuous variable, the final Model 3 showed an OR of 1.68 (1.17, 2.42) for depressive symptoms compared to those without depressive symptoms. When TyG index was categorized, participants in the highest TyG quartile had a higher adjusted OR of 1.61 (1.10, 2.35) for depressive symptoms compared to those in the lowest quartile. The generalized additive model in Table 2 further demonstrated a linear relationship between TyG index and depressive symptoms.

**TABLE 2:** Logistic regression modeling of cognitive impairment levels and TyG associations with depressive symptoms.

	Model 1		Model 2		Model 3	
	OR (95% CI)	*p*	OR (95% CI)	*p*	OR (95% CI)	*p*
Cognitive impairment level^a^ (reference = No)	2.44 (1.90, 3.13)	< 0.001	2.04 (1.49, 2.80)	< 0.001	2.64 (1.33, 5.23)	< 0.001
CERAD^b^ (reference = No)	1.78 (1.38, 2.29)	< 0.001	1.65 (1.24, 2.19)	< 0.001	2.16 (1.29, 3.63)	0.004
Animal Fluency^b^ (reference = No)	2.25 (1.75, 2.89)	< 0.001	2.25 (1.75, 2.89)	< 0.001	2.30 (1.41, 3.73)	< 0.001
Digit Symbol^b^ (reference = No)	2.41 (1.87, 3.10)	< 0.001	2.41 (1.87, 3.10)	< 0.001	2.12 (1.26, 3.54)	0.004
TYG (Continues)	1.40 (1.18, 1.67)	< 0.001	1.30 (1.08, 1.57)	0.007	1.68 (1.17, 2.42)	0.005
TYG T1	Ref		Ref		Ref	
T2	1.20 (0.83, 1.74)	0.336	1.14 (0.78, 1.68)	0.492	1.14 (0.78, 1.69)	0.494
T3	1.06 (0.72, 1.55)	0.761	0.98 (0.66, 1.46)	0.909	0.92 (0.61, 1.38)	0.687
T4	1.93 (1.36, 2.73)	< 0.001	1.67 (1.15, 2.44)	0.008	1.61 (1.10, 2.35)	0.015

*Note*: Odd ratios (ORs) for the associations between cognitive impairment levels, TyG index (as continuous and categorical variables), and depressive symptoms, under three different adjustment models. Model 1 = not adjusted, Model 2 = Model 1 + gender + age + race + education + BMI category, Model 3 = Model 2 + smoking + drinking + hypertension + diabetes + sleeping disorders + cardiovascular diseases.

^a^ The degree of cognitive impairment is the sum of the CERAD, Animal Fluency, and Digit Symbol scores, where the lowest 25th percentile is assigned a score of 1 and the other quartiles are assigned a score of 0.

^b^ The lowest 25th percentile of each test is assigned a score of 1 and the other quartiles are assigned a score of 0.

### 3.3 Subgroup Analysis and Sensitivity Analysis

Subgroup analysis was conducted to evaluate the robustness of the association between cognitive impairment and depressive symptoms. In the adjusted Model 2, stratified analysis results indicated consistent associations between cognitive impairment, TyG, and depressive symptoms across subgroups, with no significant interactions observed (Tables 3 and 4). Sensitivity analyses also supported the robustness of our findings.

**TABLE 3:** Stratified analyses of the associations between cognitive impairment level and depressive symptoms.

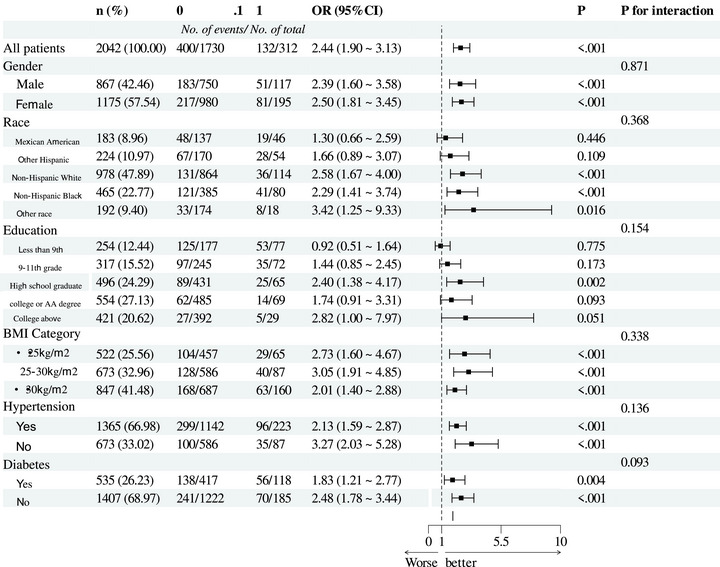

*Note*: The consistency of associations across subgroups (e.g., gender, race, BMI, and education), confirming robustness of the relationship between cognitive impairment and depression.

**TABLE 4:** Stratified analyses of the associations between TyG and depressive symptoms.

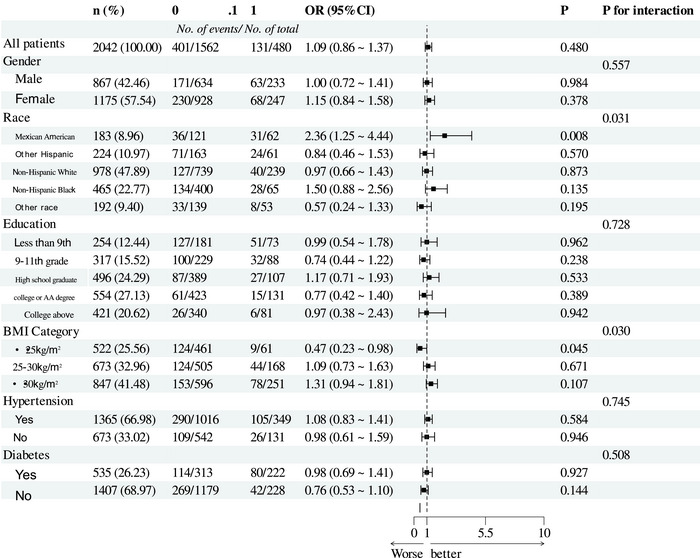

*Note*: The consistency of associations across subgroups (e.g., gender, race, BMI, and education), confirming robustness of the relationship between TyG index and depression.

## 4 Discussion

This study, based on nationally representative cross‐sectional data from the NHANES, investigated the associations between cognitive impairment, TyG index, and depressive symptoms in older adults. The main findings indicate that cognitive impairment and higher TyG index levels are significantly associated with depressive symptoms in the elderly. These results remained consistent across various stratification and sensitivity analyses, suggesting that a higher TyG index could be an effective indicator for assessing depression risk. To our knowledge, this is the first study to focus on the connection between cognitive function, TyG index, and depression in older adults.

The positive correlation between cognitive impairment and depressive symptoms aligns with previous research. Numerous population‐based studies have revealed the complex interplay between late‐life depression and cognitive decline (Hu et al. 2019; Wei et al. 2019), with conditions like MCI and dementia being closely linked to an increased risk of depression (Kopchak and Pulyk 2017; Spira et al. 2012; Mirza et al. 2016). In fact, some researchers suggest that poor cognitive performance may indicate early dementia (Mirza et al. 2016). Our study found that the risk of depression in elderly individuals with cognitive impairment was significantly higher (OR = 2.64, 95% CI: 1.33–5.23), consistent with prior findings (Muhammad and Meher 2021). Recent meta‐analytic evidence further supports this link, showing that individuals with cognitive decline had significantly higher TyG index levels than cognitively normal individuals, and that each 1‐unit increase in TyG was associated with a 2.86‐fold increase in odds of cognitive decline (95% CI: 1.49–5.50) (Ghondaghsaz et al. 2024). Additionally, a separate meta‐analysis confirmed that higher TyG index is also associated with increased risk of depression and suicidal ideation (Behnoush et al. 2024), reinforcing the clinical relevance of TyG as a potential marker for both cognitive and affective dysfunction in the elderly.

Insulin resistance is increasingly recognized not only as a central component of metabolic syndrome and cardiovascular disorders but also as a critical contributor to cognitive impairment and depression. Several biological mechanisms may underlie these associations. First, insulin resistance promotes neuroinflammation, characterized by chronic low‐grade inflammatory responses, elevating pro‐inflammatory cytokines (such as interleukin‐6 [IL‐6] and tumor necrosis factor alpha [TNF‐α]), which impair neuroplasticity and enhance neuronal vulnerability (Leonard and Wegener 2020). Second, insulin resistance is associated with cerebrovascular dysfunction, potentially leading to microvascular insufficiency, cerebral hypoperfusion, and subsequent impairment in cognitive function and emotional regulation (Nam et al. 2020). Lastly, insulin signaling within the central nervous system has neuroprotective roles, and disruption of these insulin‐signaling pathways due to insulin resistance may negatively affect neuroplasticity, particularly within hippocampal and prefrontal cortical regions, thereby exacerbating cognitive decline and heightening susceptibility to depression.

The association between cognitive impairment and depressive symptoms encompasses complex biological, psychological, and sociological factors. These factors collectively explain why cognitive impairment is a significant risk factor for depression in the elderly (Culpepper et al. 2017). First, cognitive impairment may directly affect brain structure and function, particularly in areas such as the prefrontal cortex and hippocampus, which are closely related to emotion regulation. Damage or functional decline in these areas may lead to imbalanced emotional regulation mechanisms, increasing the risk of depression. Cognitive impairment is also associated with changes in neurotransmitters, such as dopamine and serotonin imbalances, which may promote depressive symptoms. Additionally, social and psychological factors cannot be ignored. Elderly individuals with cognitive impairment may feel socially isolated or functionally impaired due to their symptoms, exacerbating the risk of depression (Muhammad and Meher 2021). The link between depression and cognitive impairment is also influenced by various neurobiological factors, such as genetic factors like the apolipoprotein E epsilon 4 allele (APOE‐e4) allele associated with AD and depression risk (Wang et al. 2019) and the roles of brain‐derived neurotrophic factor (BDNF) and transforming growth factor‐beta 1 (TGF‐β1) in the pathophysiological process (Borroni et al. 2009). Inflammation also plays a central role: Recent findings suggest that chronic low‐grade inflammation and metabolic dysfunction may converge to influence mood and cognition. In particular, a recent study demonstrated that the TyG index—a surrogate for insulin resistance—was associated with depressive symptoms and elevated VEGF levels, especially in older women (Mancinetti et al. 2025). This sex‐specific inflammatory signature suggests that metabolic‐inflammation pathways, including markers like VEGF, MCP‐1, and CRP, may underlie the TyG–depression link, reinforcing the need for sex‐stratified and biomarker‐informed approaches in late‐life depression research (Mancinetti et al. 2025). Therefore, early cognitive function assessments in the elderly to detect potential cognitive impairments are crucial for preventing and treating depression.

The TyG index reflects insulin resistance and is associated with various health issues, including cardiovascular diseases, metabolic syndrome, and depression (Zhang et al. 2017; Bornfeldt and Tabas 2011; Zhu et al. 2020). Insulin resistance may lead to increased inflammation and oxidative stress in the brain, which are directly linked to the development of depression (Nam et al. 2020). A high TyG index may indicate not only insulin resistance but also a burden of other health conditions, which may collectively trigger depressive symptoms through physiological and psychological pathways.

Our findings have important clinical implications. First, routine cognitive assessments in elderly populations could help identify those at higher risk for depressive symptoms, enabling timely preventive interventions. Early identification of cognitive impairment allows healthcare providers to initiate targeted mental health support and cognitive interventions, potentially mitigating the progression of depressive symptoms. Second, incorporating the TyG index as a simple, cost‐effective marker of insulin resistance into regular clinical screening protocols may help clinicians identify older adults at increased risk for depression due to metabolic disturbances. By recognizing elevated TyG index levels early, healthcare providers could recommend appropriate lifestyle modifications (e.g., dietary changes, physical activity) or medical treatments aimed at improving insulin sensitivity and potentially reducing depressive symptoms. Overall, our results suggest that integrating cognitive function tests and TyG index measurements into routine clinical practice could enhance depression screening accuracy, inform comprehensive geriatric assessments, and ultimately improve mental health outcomes among elderly populations.

### 4.1 Strengths and Limitations

This study has several strengths. First, it focuses on the relationship between cognitive function, TyG index, and depression in older adults. Second, it uses a complex multistage probability sampling design to select NHANES data, representing a noninstitutionalized civilian population, ensuring high data quality and reliability when generalizing the results to the entire US noninstitutionalized population. Third, the study controlled for numerous confounding factors, including sociodemographic characteristics, hypertension, diabetes, sleep disorders, and other comorbidities, using three models to validate the consistency of the findings. Fourth, the cognitive impairment scores were derived from the sum of three cognitive tests (CERAD, Animal Fluency, and DSST), objectively illustrating the relevant connections with elderly depression. These findings have additional public health implications for preventing depression in older adults.

This study also has some limitations. First, due to the cross‐sectional design, our findings cannot infer cause‐and‐effect relationships between cognitive impairment, TyG index, and depressive symptoms in older adults. Prospective cohort or interventional studies are required to explicitly test causality and further clarify the temporal sequence and underlying biological mechanisms of these associations. Second, due to database limitations, the results primarily apply to the US population, and these findings may not be directly generalizable to other ethnic groups and regions. Our findings are primarily applicable to the US population. Given differences across cultural, socioeconomic, and healthcare contexts, the generalizability of our results to other populations may be limited. Future studies should validate these findings in diverse ethnic and international populations to better understand how contextual factors influence these associations. Third, PHQ‐9 was used to diagnose depression, a self‐reported assessment without clinical confirmation. Although this method is widely used in clinical and epidemiological studies and has been validated with high sensitivity and specificity, depression encompasses mild, moderate, and severe forms, which may relate differently to cognitive impairment and TyG index. Although PHQ‐9 is widely validated and used extensively in epidemiological studies, as a self‐report measure, it may be subject to recall and social desirability biases. Future research should integrate clinician‐confirmed diagnostic assessments alongside self‐reports to strengthen the reliability of the findings. Fourth, the relationship between antidepressant use and oxidative stress was not considered due to data limitations, excluding potential confounding factors related to medication use. Due to limitations in the NHANES database regarding antidepressant medication use, our study could not account for the potential confounding effects of antidepressants on cognitive function, metabolic indices, and depressive symptoms. Future research should systematically collect medication usage data to better control for these potential confounders. Fifth, physical activity data in NHANES are self‐reported and subject to recall bias and social desirability bias. In addition, the high proportion of missing physical activity data in the sample may lead to reduced statistical power and potential bias. Therefore, physical activity was not included as a covariate in our final analyses. Future studies should aim to incorporate objective measures of physical activity (e.g., accelerometers or activity trackers) to minimize bias and improve the reliability of findings. Finally the NHANES database does not include a direct diagnosis of dementia. Although cognitive functioning was assessed by several standardized tests, there were no specific variables to identify participants with dementia. Therefore, we were unable to exclude people with dementia‐related depressive symptoms from our analyses. This is a limitation of our study, and future research needs more comprehensive data on dementia diagnoses to explore the impact of dementia‐related depressive symptoms. The absence of specific dementia diagnosis variables in the NHANES database limits our ability to differentiate depressive symptoms directly related to dementia. Future studies should incorporate detailed diagnostic criteria for dementia to more accurately delineate its impact on depression and cognitive impairment. The wide CI observed in the association between depressive symptoms and cognitive impairment (OR = 2.64, 95% CI: 1.33–5.23) suggests a limited number of participants in this subgroup after weighting, leading to sparse‐data uncertainty. Future studies with larger or pooled datasets are warranted to validate this association. Additionally, logistic regression may not fully capture complex, nonlinear relationships or effectively handle highly correlated variables, potentially resulting in unstable estimates or omitted variable interactions. Although our logistic regression models provided valuable explanatory insights, future research should consider employing machine learning techniques, such as random forest classifiers or gradient boosting models, to explore possible nonlinear relationships and improve robustness of feature selection.

## 5 Conclusions

This study demonstrates that, after adjusting for covariates, higher TyG index levels and cognitive impairments (including learning and memory, executive function, attention, and processing speed) are positively associated with depression. These findings suggest that the TyG index and cognitive impairment may be independent secondary predictors of depression development.

## Author Contributions

**Qinghua Guo**: conceptualization, investigation, funding acquisition, writing – original draft, validation, methodology, software, data curation, resources, supervision, project administration, formal analysis, visualization, writing – review and editing. **Chao Chen**: conceptualization, methodology. **Libo Guo**: conceptualization. **Yong Wang**: conceptualization. **Shaomei Shang**: conceptualization, methodology.

## Ethics Statement

This research analyzed de‐identified information downloaded from the National Health and Nutrition Examination Survey public database. The National Center for Health Statistics Ethics Review Committee granted ethics approval. All methods were carried out in accordance with relevant guidelines and regulations (declaration of Helsinki). All individuals provided written informed consent before participating in the study (https://www.cdc.gov/nchs/nhanes/irba98.htm).

## Consent

The authors have nothing to report.

## Conflicts of Interest

The authors declare no conflicts of interest.

## Peer Review

The peer review history for this article is available at https://publons.com/publon/10.1002/brb3.70824

## Data Availability

The datasets generated and analyzed in the current study are available at NHANES website (https://www.cdc.gov/nchs/nhanes/index.htm). The data that support the findings of this study are available from the corresponding author upon reasonable request.
